# High-resolution computed tomography illustrating pulmonary lymphangitic carcinomatosis in a patient with advanced pancreatic cancer: a case report

**DOI:** 10.1186/1757-1626-2-7428

**Published:** 2009-05-12

**Authors:** Liao Wan-Hsiu, Lin Sheng-Hsiang, Wu Tsu-Tuan

**Affiliations:** 1 Department of Family Medicine, Taipei County HospitalTaipei CountyTaiwan (R.O.C.); 2 Department of Internal Medicine, Taipei County HospitalTaipei CountyTaiwan (R.O.C.)

## Abstract

We present a case of advanced pancreatic cancer with diffuse pulmonary interstitial infiltrates and dyspnea in a 61-year-old Asian Taiwanese female. Although surgical lung biopsy is the diagnostic gold standard in most interstitial lung disease, it frequently leads to complications in sick patients. Based on the overall clinico-radiologic correlation, a diagnosis of pulmonary lymphangitic carcinomatosis was supported by the characteristic findings in high-resolution computed tomography.

## Introduction

Pulmonary complications are frequently countered in patients with advanced cancer. Although less frequent, the most challenging issue is the so-called “diffuse interstitial infiltrates” on chest radiography. In these patients, the differential diagnosis of various etiologies is difficult. However, the characteristic findings in high-resolution computed tomography (HRCT) are helpful in the diagnosis of lymphangitic carcinomatosis.

## Case presentation

**Figure 1.**
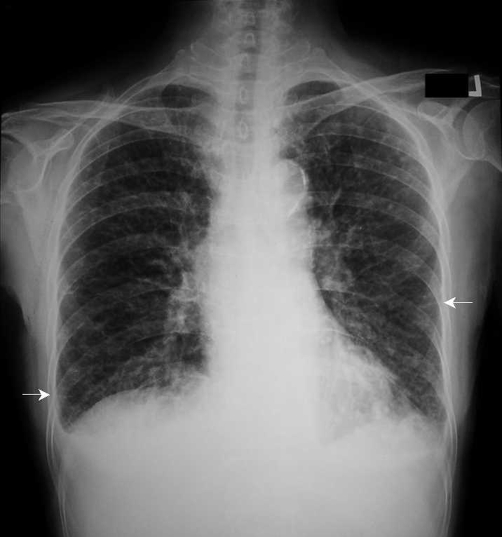
Chest radiograph showing diffuse interstitial pulmonary infiltrates with Kerley B lines (arrows) and pleural effusion.

**Figure 2.**
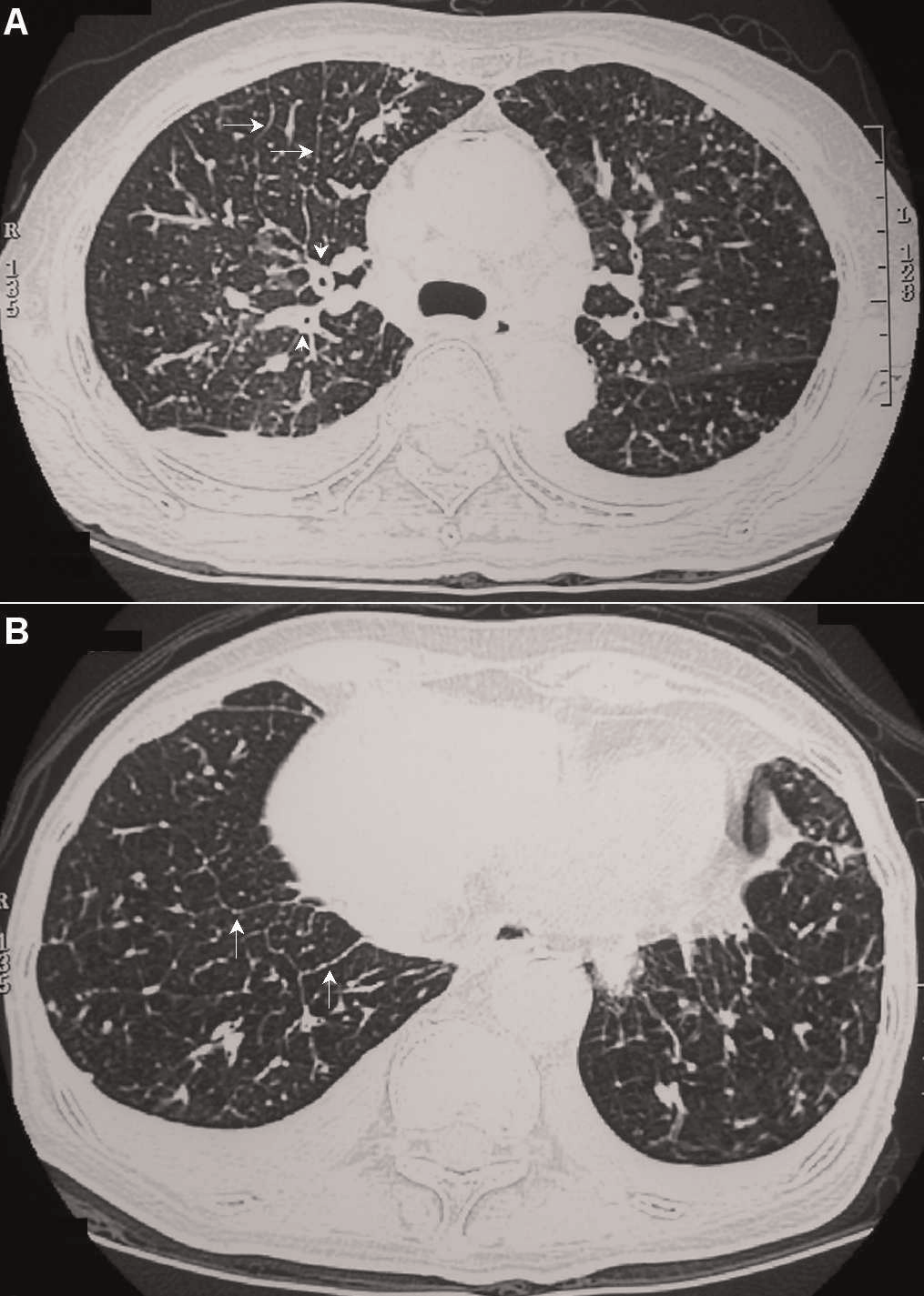
High-resolution chest computed tomography showing **(A)** Peribronchovascular thickening (arrowheads), irregular thickening of interlobular septa (arrows) and bilateral small amount pleural effusion **(B)** Interlobular septal thickening forming polygonal arcades (arrow) with prominence of the centrilobular bronchovascular bundle (a central dot in a polygon).

A 61-year-old Asian Taiwanese woman having pancreatic cancer with liver and left adrenal metastases presented with progressive dyspnea for one month. Chest examination revealed crackles at the bases. Cardiovascular examination showed no jugular venous distension and normal heart sounds. An echocardiogram showed normal left ventricular function. A chest radiograph (Figure 1) revealed diffuse reticular opacities and bilateral small amount pleural effusion. HRCT of the lung (Figure 2) showed thickening of peribronchovascular interstitium, interlobular septa, and centrilobular regions. Cytological examination of the pleural fluid revealed adenocarcinoma, most suggestive of a primary origin in the pancreas. However, there was no response to chemotherapy with gemcitabine and she passed away one month later.

## Discussion

The differential diagnosis of dyspnea in a patient with advanced cancer and diffuse interstitial pulmonary infiltrates in chest radiograph is extensive, including viral pneumonia [1], hydrostatic pulmonary interstitial edema [2], chemotherapeutic drugs-induced lung toxicity [3], and lymphangitic carcinomatosis [4]. Since lung biopsies carry a high risk for complications in these patients [5], the characteristic findings of lymphangitic carcinomatosis on HRCT make great contributions to the differential diagnosis [6].

## Conclusion

In cases with advanced cancer and diffuse interstitial pulmonary infiltrates, HRCT may make contributions to the differential diagnosis, especially while lymphangitic carcinomaotsis is suspected.

## List of abbreviations

HRCT: high-resolution computed tomography
